# Supplementary material for: GenoPipe: identifying the genotype of origin within (epi)genomic datasets
Source: Nucleic Acids Res. 2023 Nov 2;51(22):12054–68. doi: 10.1093/nar/gkad950 (PMC10711449; doi:10.1093/nar/gkad950)
Supplement: gkad950_Supplemental_Files [file gkad950_supplemental_files.zip › Supplemental_Figures.pdf]

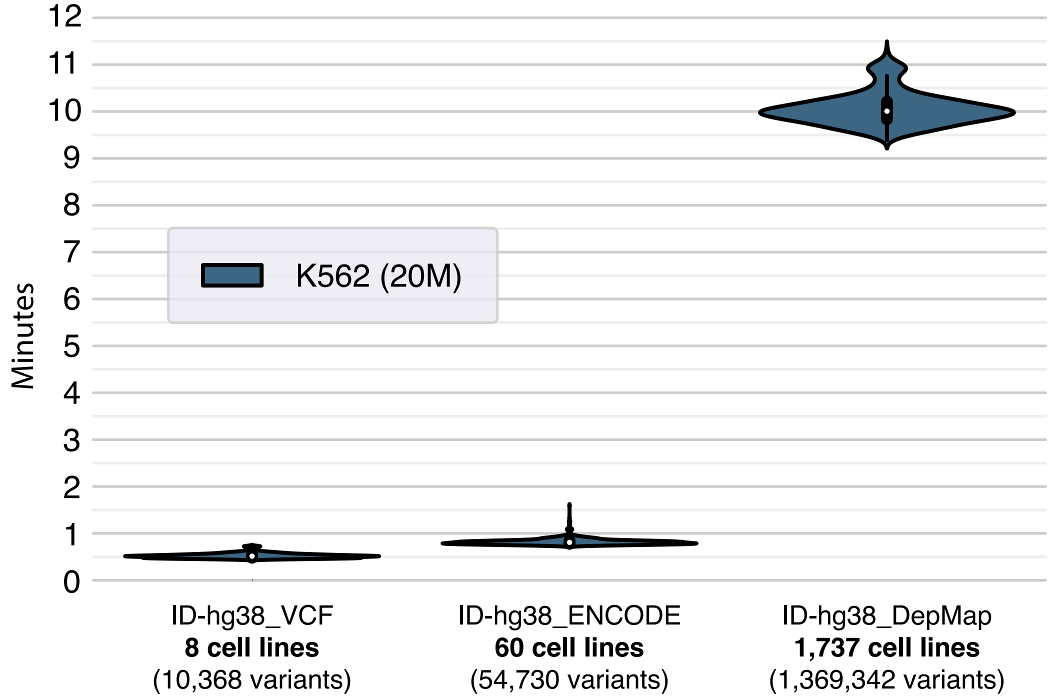

Supplementary Figure 1. The StrainID module performance scales with the number of variants in the VCF database. Using the simulated K562 datasets with 20 million reads, we measured the runtime for running StrainID against three databases with an increasing number of cell line references.

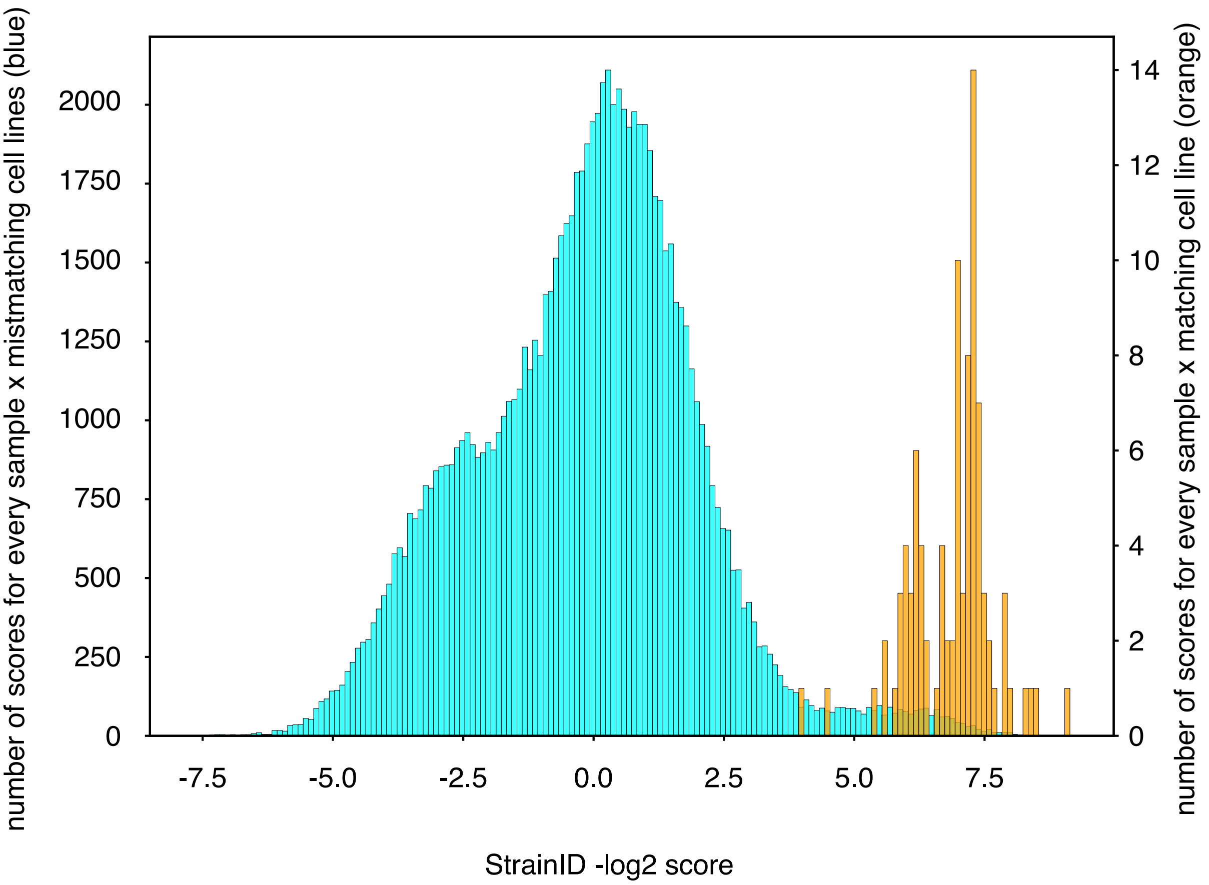

Supplementary Figure 2. StrainID scores for the matching cell line show good separation from the scores from other cell lines. 100 TF-ChIP-seq datasets from ENCODE were run through StrainID using the hg38_DepMap reference set of over 1,000 cell lines. The scores matching and not matching the “correct” or ENCODE labelled cell line were plotted on top of each other as histograms in the figure with orange showing the matching StrainID scores and blue showing the scores from the other cell lines.
